# An eight-year retrospective study on the clinical outcomes of laser surface-treated implants

**DOI:** 10.1186/s40729-024-00558-7

**Published:** 2024-08-05

**Authors:** Richard Leesungbok, Sung Ok Hong, Suk-Won Lee, Phyo Ei Ei Htay, Joseph Junesirk Choi, Jung Jin Park

**Affiliations:** 1 Department of Biomaterials and Prosthodontics, Kyung Hee University Dental Hospital at Gangdong, Kyung Hee University College of Dentistry, Dongnamro 892, Gangdong-Gu, Seoul, 05278 Republic of Korea; 2 Department of Oral and Maxillofacial Surgery, Kyung Hee University Dental Hospital at Gangdong, Kyung Hee University College of Dentistry, Dongnamro 892, Gangdong-Gu, Seoul, 05278 Republic of Korea; 3 Department of Orthodontics, Dental Hospital, Kyung Hee University Hospital at Gangdong, Seoul, Republic of Korea; 4 Professor Emeritus, Kyung Hee University School of Dentistry, Seoul, South Korea

**Keywords:** Laser treated implant, ISQ value, Insertion torque value, Marginal bone loss, Immediate loading

## Abstract

**Purpose:**

To retrospectively evaluate peri-implant bone loss and health status associated with the long-term use of laser surface-treated implants.

**Methods:**

For control study, total of 23 titanium ASTM F136 grade 23 implants were placed in the edentulous molar area of the mandible. When the Implant Stability Quotient (ISQ) ≥ 70 and insertion torque value (ITV) ≥ 35–50 Ncm at the insertion site, an immediate provisional restoration was connected to the implant within a week after surgery. The definitive restorations were placed 2 months after surgery for all implants. 13 implants were immediately loaded, while 10 implants were conventionally loaded. For comparative study, Radiographs were taken from third years for and then annually for the subsequent eight years to monitor marginal bone loss.

**Results:**

After eight year of implant installation, the average change in vertical bone loss was 0.009 mm (P < 0.001), while the average change in horizontal bone loss 8 year after implant placement was 0.026 mm (P < 0.001). The mean marginal bone loss was < 0.2 mm on average.

**Conclusions:**

In this retrospective study, laser-treated implants exhibit a low rate of bone absorption around the implants.

## Introduction

The primary objectives of implant surface treatment encompass the following: increasing the surface area to achieve a stronger initial mechanical fixation between the implant and bone upon insertion [1], maintaining an effective blood clot-retaining surface structure [2], and promoting the bone healing process [3]. In particular, the SLActive technique involves creating surface roughness using a large grit with a diameter range of 250–500 µm after sandblasting and etching with hydrochloric and sulfuric acids, followed by a nitrogen wash [4].

This process results in the formation of a hydroxyl layer with a high surface energy when in contact with water, thereby facilitating optimal interaction between the implant and surrounding factors [4]. The activated surface is preserved and stored in a physiological saline solution commonly used in dental clinics [5], combining chlorine ions, anions, and hydroxyl ions to safeguard the activated surface from exposure to air and prevent hydrocarbon binding [6–8]. Based on previous studies, it is evident that these surface properties significantly enhance bone-to-implant contact, and ultimately accelerate the healing process of osseointegration during the early stages. In effect, this leads to enhanced stability of the implant and promotes healing during the critical early stages [9–11].

According to a recent study, laser-treated surface implants help improve the osseointegration process [13]. This unique surface treatment method effectively prevents contamination from external factors and maintains a high degree of surface purity, resulting in excellent surface roughness. In other words, the entire laser-treated surface of the implant possesses a pure and uncontaminated porous structure. Along with increasing surface roughness, this configuration also augments the strength of osseointegration [14, 15].

ASTM F136 is a standard specification for titanium alloy for surgical implant applications. Grade 23 refers to a specific type of titanium alloy, also known as Ti-6Al-4 V ELI (Extra Low Interstitial). This alloy is commonly used in medical implants due to its excellent biocompatibility, corrosion resistance, and high strength. Implants made from ASTM F136 Grade 23 titanium are often used in orthopedic, dental, and prosthetic applications. They are known for their ability to integrate well with the human body, reducing the risk of rejection or adverse reactions.

Implant loading protocols were defined as follows according to Morton et al. [16]

- Immediate loading: Dental implants are connected to a prosthesis in occlusion with the opposing arch within 1 week subsequent to implant placement.
- Early loading: Dental implants are connected to the prosthesis between 1 week and 2 months after implant placement.
- Conventional loading: Dental implants are allowed a healing period of more than 2 months after implant placement with no connection of the prosthesis.

Nevertheless, there has been a noticeable absence of clinical studies concerning the immediate and early loading of implants with laser-treated surfaces, despite their demonstrated excellent osseointegration in animal studies [17].Therefore, our primary objective was to apply laser treatment to an implant surfaces and conduct a clinical trial to investigate the feasibility and safety of loading them, contingent on the initial stability following implant placement in the human jaw [18].

The purpose of the study was to demonstrate the performance of laser-treated implants through longitudinal observation. By comparing the results of the control group, which was based on a previously published article, with the results of the comparison group using current data, the researchers aimed to evaluate the effectiveness of laser-treated implants over a longer period of time. We hypothesized that immediate loading could be safely applied in implants with laser-treated surface even by longitudinal observation.

## Materials and methods

### Inclusion criteria

1. Adults over 18 years of age who have completed the growth of the jawbone and voluntarily consented in writing to the clinical trial
2. Those with natural tooth loss
3. Those who do not have severe maxillary and mandibular relationship dissonance
4. Those who have sufficient available bones vertically, mesiodistal, and buccal
5. Those who do not have masticatory disorders in other molars, premolars, and canines
6. Those who do not need maxillary sinus elevation and bone graft for maxillary teeth

### Exclusion criteria

1. People with bone disease
2. Those with maxillary sinus disease
3. Those with metabolic diseases such as thyroid and diabetes
4. Those with bleeding disorders or those requiring anticoagulants
5. Those with systemic disease that makes extraction difficult
6. Mental illness or suspected mental illness
7. Those who have difficulties in implant surgery such as tooth grinding or lack of space for prostheses
8. Persons with a disability in temporomandibular joint disease
9. Pregnant women and the elderly
10. Others who are inappropriate to participate in clinical trials in the judgment of the clinical trial director because they may affect the clinical trial results or ethically

### Materials

The implants were constructed from titanium ASTM F136 grade 23, featuring a screw-shaped design, and measured 4.2 mm in diameter and 10 mm in length (CSM; Daegu, Republic of Korea) LC3FM10 (Submerged I System).

The laser treatment performed directly on the machined titanium surface with an Nd:YAG laser (Jenoptic Laser Optik, Jena, Germany), with linear motion,at a power setting of 7 W, representing energy and frequency levels of 120 mJ and 50 Hz; this is the same method according to Cho et al. [17].

### Control and comparative group

**Fig. 1:**
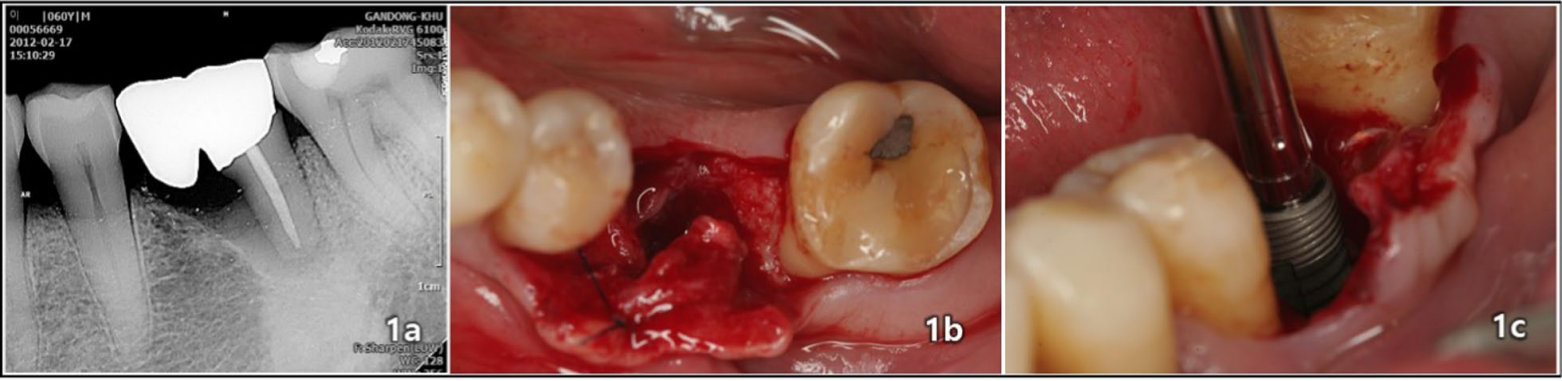
A 60-year-old male patient visited the hospital with severe mobility on the lower left first molar: **a** Periapical radiographic image during the initial examination; **b** immediately after the extraction of the lower left first molar; and **c** immediate implantation of the laser treated surface implant (4.2 diameter, 10 mm length, CSM, Seoul, Republic of Korea)

**Fig. 2:**
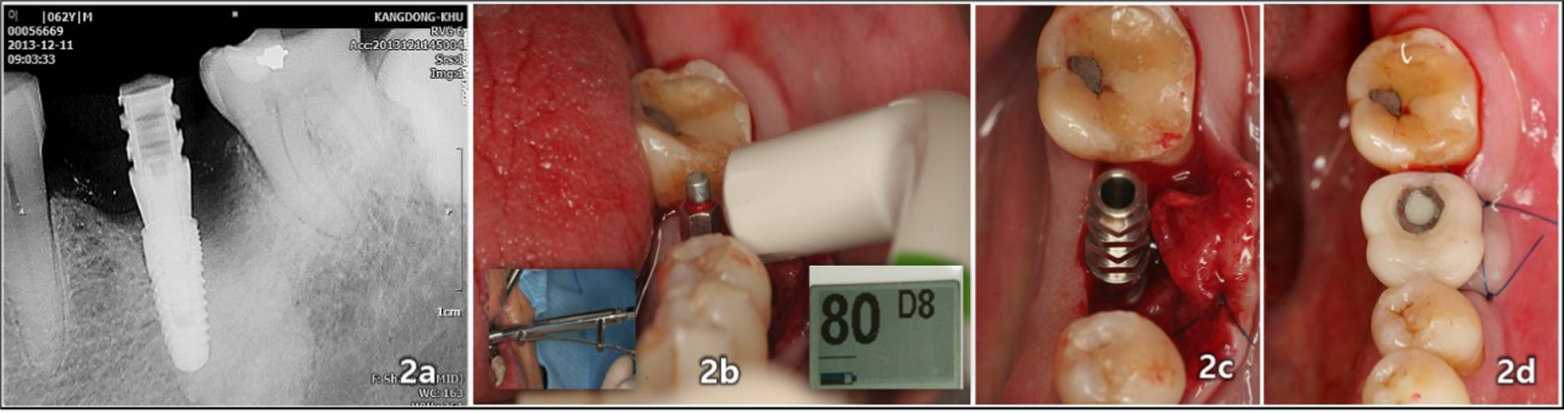
Immediate loading with immediate provisional restoration involved the following steps: **a** A periapical radiographic image was taken immediately after the placement of the laser-treated surface implant (4.2 diameter, 10 mm length, CSM, Seoul, Republic of Korea) with an immediate provisional restoration on December 11, 2013. **b** The insertion torque value (35 NCm) was measured using a mechanical torque gauge, and primary stability (ISQ 80) was assessed with OsstellTM Mentor® (Integration Diagnosis, Göteborg, Sweden). **c** A temporary abutment was connected for the purpose of facilitating immediate loading to the implant. **d** The process was completed with the screw-fastening of a temporary acrylic resin restoration, enabling immediate functional occlusal loading to the implant

**Fig. 3:**
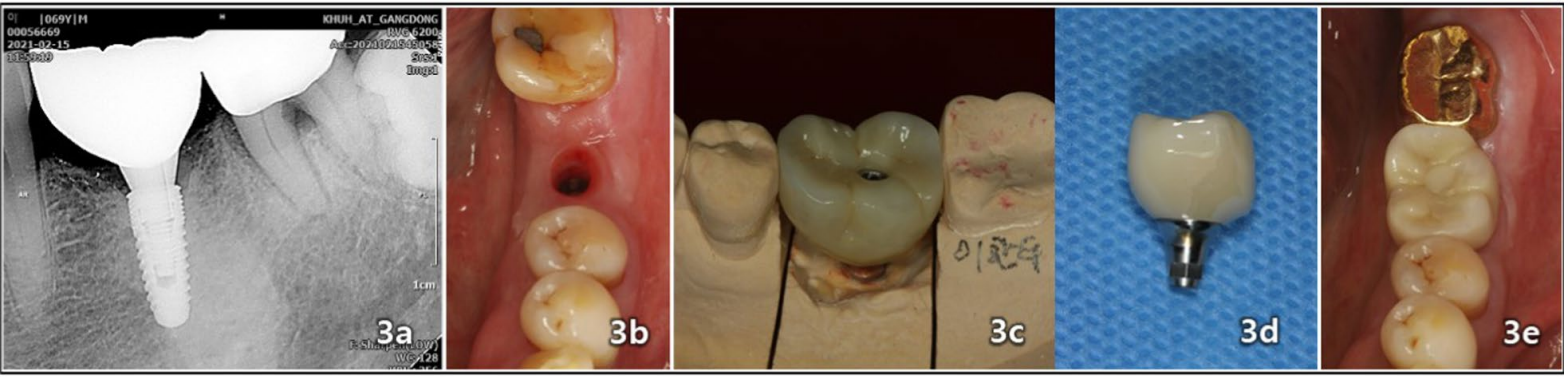
Insertion of the final restoration and clinical follow-up included the following: **a** A nine-year follow-up involved a periapical radiographic image of the operation site after the placement of the Monolithic Zirconia crown as the definitive restoration. **b** This was conducted prior to the insertion of the definitive implant restoration on February 15, 2014. **c**, **d** The definitive restoration was accomplished with an implant-fixed, screw-fastened Monolithic Zirconia crown. **e** One year after the insertion of the definitive implant restoration, a follow-up was conducted

**Fig. 4:**
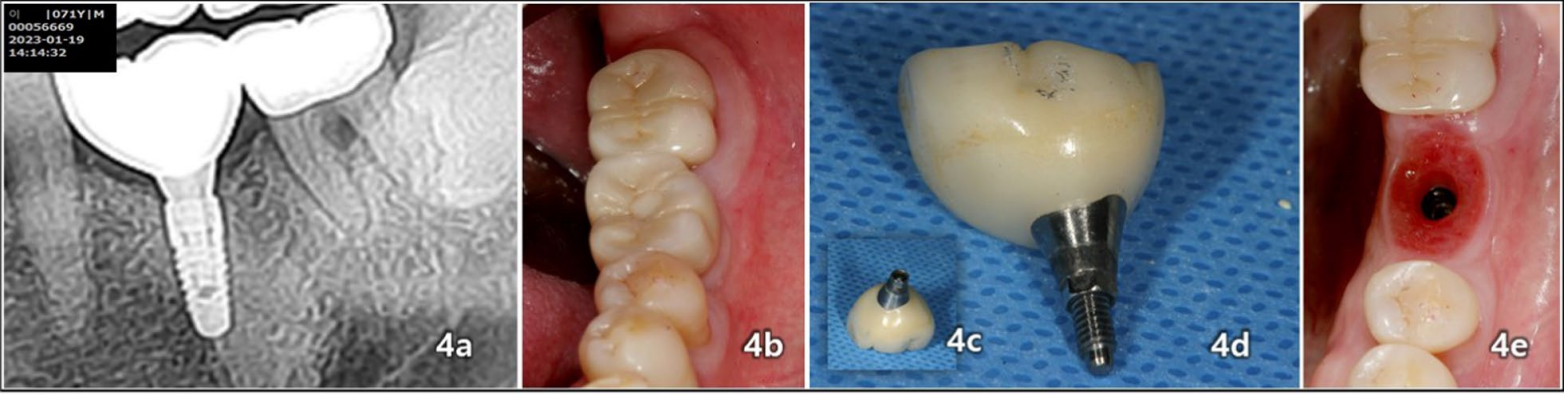
A 10-year clinical follow-up after immediate functional loading of the implant revealed the following: **a** A 10-year follow-up involving the periapical radiographic image on the operation site taken on January 19, 2023. **b** An intraoral occlusal view of implant-fixed screw-fastening Monolithic Zirconia crown on the lower left first molar site. **c**, **d**, **e** Examination of the crown and mucosae of the transmucosal part after removal of the definitive Zirconia crown on the operation site. Notably, there was an absence of plaque or debris, and no signs of an inflammatory response were observed

It is possible to use the results of a previous study as a historical control group and compare them with a comparative group that tracks the same patients for a long-term period. This method can be useful in research for assessing changes and trends. The data collected from the previous study is reliable, provides standardized information about the same patient group, and aligns with the current research questions and study objectives. Therefore, using the previous study as a control group to compare bone changes after finishing the first project years is a valid approach (Figs. 1, 2, 3, 4).

This study compares the outcomes of two groups: a control group, also known as a "historical control group” and a comparison group that utilized the latest clinical data from KHNMC 2021-01-052-001, collected four years later for the same patients.

The control group study, as mentioned earlier, focused on evaluating the clinical outcomes of immediately and early loaded implants with laser-treated surfaces over a three-year period, which was established based on a previously published article titled "Clinical outcome of immediately and early loaded implants with laser-treated surface: a 3-year retrospective study" [18].

The control group consisted of 15 patients who willingly participated in the clinical trial. The trial was successfully completed without any dropouts among the participants following the implant surgery. In total, 23 implants were placed in these 15 patients, with 13 implants being immediately loaded and the remaining 10 implants conventionally loaded.

**Fig. 5:**
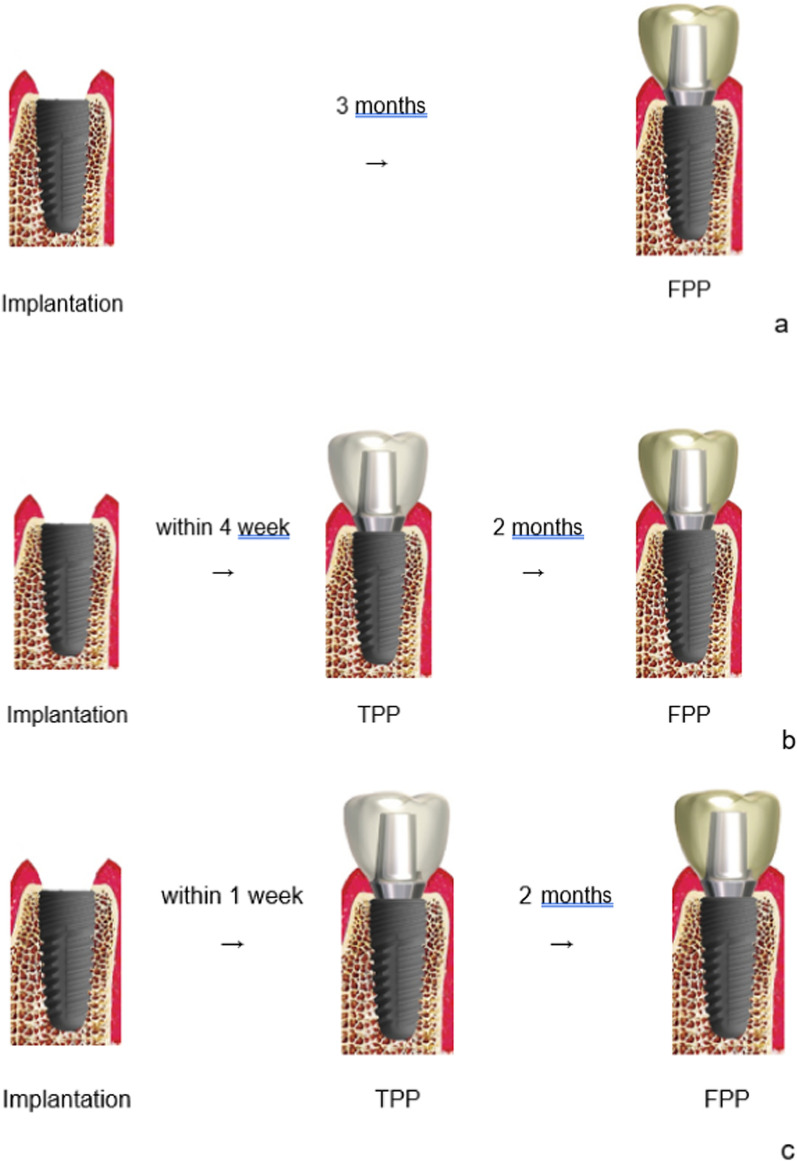
**a** Conventional loading: Implant Stability Quotient (ISQ < 60) and the insertion torque is less than 25 Ncm. **b** Early loading: ISQ ≥ 60 and the insertion torque is 25 ~ 35 Ncm. **c** Immediate loading: ISQ ≥ 70 and insertion torque is 35 ~ 50 Ncm

A comparative table has been provided to clearly display the number of implants that were immediately loaded and conventionally loaded (Fig. 5).

The inclusion of early loading protocols within the definition of "immediate" helps capture a wider range of loading timelines by expanding the scope of the term. By considering early loading as part of the immediate loading category, we are able to incorporate loading protocols that involve a four-week period into our study.

Traditionally, the term "immediate loading" referred only to the placement of the final prosthesis onto the implant within a short timeframe, typically one week after installation. However, by broadening the definition, we now include protocols where a temporary prosthesis is applied to the implant four weeks post-installation, with the final prosthesis attached two months later.

Including early loading protocols within the immediate loading category allows us to examine the effects and outcomes of loading dental implants at different timepoints during the early healing period. It enables us to assess the impact of loading timelines that fall within the four-week timeframe, thereby capturing a wider range of clinical scenarios.

This expanded definition ensures that we consider the various approaches used in practice and accounts for the individualized needs of patients. By encompassing both immediate loading and early loading protocols within the term "immediate," we aim to provide a more comprehensive understanding of the implications of loading timelines on implant success and long-term treatment outcomes.

Prior to surgery, all patients received prophylactic antibiotics two hours in advance, and rinsed their mouths with a 0.12% chlorhexidine solution for one minute. The procedure involved elevating a full-thickness flap under local anesthesia.

### Loading protocol in the previous control study

The implants were inserted into the edentulous molar area of the mandible, following protocols as follows:

1. When the Implant Stability Quotient (ISQ) ≥ 70 and insertion torque value (ITV) ≥ 35–50 Ncm at the insertion site, a provisional restoration was connected to the implant within a week after surgery. The final restoration was placed two months after surgery for immediate loading.
2. In cases where ISQ is between 60–70 and ITV is 25–35 Ncm at the implant insertion, an impression was taken within two weeks after surgery, with the final restoration placed two months post-op for early loading

### Bone measurements conducted in this comparative study

Digital bisecting radiographs were obtained annually for up to eight years, then processed using a software program. First bone-implant contact (FBIC) was measured on the mesial and distal planes of the implant. For calibration purposes, a known pitch distance between the implant threads was used. The FBIC was measured for all 23 implants at baseline. Vertical dimension (VD) and horizontal dimension (HD) bone loss/gain were calculated as the difference in bone level (BL) at baseline and the FBIC at a certain endpoint in each period (4, 5, 6, 7, and 8 years) for the comparative study. An vertical and horizontal bone loss ≤ 1.5 mm were indicative of success.

### Statistical analysis

The data are presented as mean ± standard deviation, and statistical analysis was performed using repeated-measures ANOVA to identify changes in marginal bone loss over time. All data were analyzed using a statistical software (SPSS ver 25.0; IBM, Chicago, IL, USA) (α = 0.05). First, normal distribution of the data was investigated using the Shapiro–Wilk test, and because a normal distribution was not achieved, the difference between the groups was verified using the Mann–Whitney U test. The Friedman’s test was used to confirm these changes.

The sample size was determined using a One-Sample Design. The calculations assumed that there was nearly no significant difference between the normally distributed groups, with a test of concordance between 2.5 Ncm SD(δ) and 3.5 Ncm SD(μ). The significance level of α was set at 0.05, and the β value was set at 0.05, yielding a 95% power level. The magnitude (ε) of the effect was ε = μ – δ = 1SD. A standard deviation of σ = 1 was employed based on the standard normal distribution. As a result, the appropriate number of subjects required to confirm the treatment effect of 1SD was calculated using the following formula: from 3.1.2 of test for equality [19];

$${\text{n}}\, = \,\left( {{\text{Z}}\alpha /{2}\, + \,{\text{Z}}\beta } \right){2 }\sigma {2}/\varepsilon {2}\, = \,\left( {{1}.{96}\, + \,{1}.{64}} \right){2}/{12}\, = \,{12}.{99}.$$

At the control (previous) study, the historical control group, we opted for 95% power to enhance reliability.

## Results

The average change in bone loss in the vertical direction within the first year following implant installation was ΔVD 0.009 mm (P < 0.001). Notably, between the first and second years, as well as between the third and fourth years, the average change in the VD value over one year was negative. This was ascribed to specific clinical cases displaying bone growth in the vertical direction (as illustrated Table 1).

**Table 1:** Average change in bone loss over 1 to 8 years by location (P < 0.001)

	ΔHD1	ΔVD1	ΔHD2	ΔVD2
1Y	− 0.06	− 0.11	0.01	0.06
2Y	0.19	− 0.01	–0.01	–0.10
3Y	− 0.01	0.03	− 0.02	− 0.02
4Y	− 0.04	− 0.03	− 0.03	− 0.10
5Y	0.04	0.02	0.02	0.04
6Y	0.03	0.05	0.12	0.14
7Y	0.03	0.07	0.02	0.08
8Y	− 0.02	0.00	0.05	− 0.05

Y, year; ΔHD1, Horizontal Bone loss (Mesial); ΔHD2, Horizontal Bone loss (Distal); ΔVD1, Vertical Bone loss (Mesial); ΔVD2, Vertical Bone loss (Distal)

Similarly, the average change in bone loss in the horizontal direction within the first year after implant placement was ΔHD 0.026 mm (P < 0.001). Between the second and third years and between the third and fourth years, the average change in the HD value over one year was also negative. Again, this was ascribed to specific clinical cases displaying bone growth in the horizontal direction (as demonstrated Table 1).

**Fig. 6:**
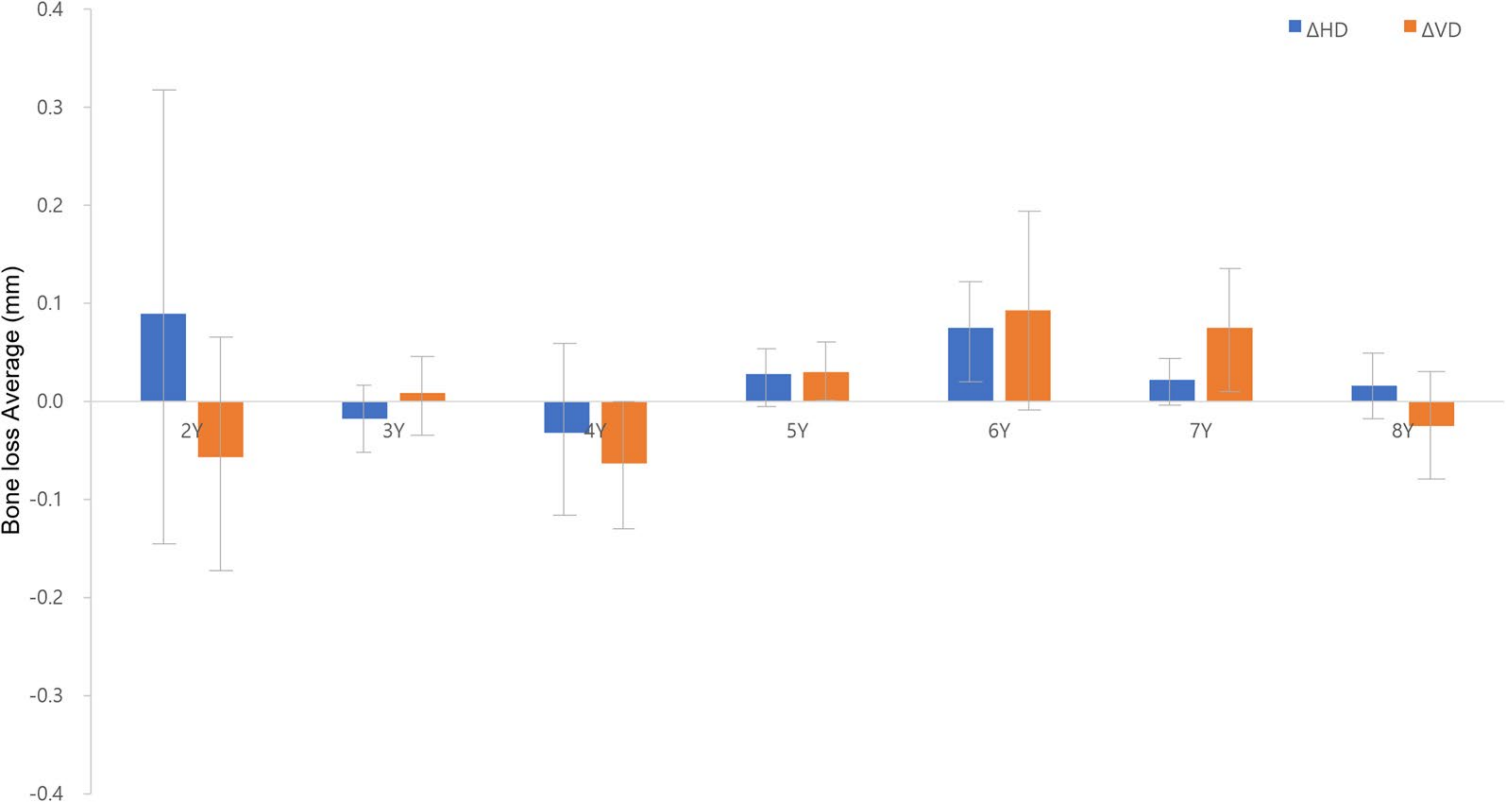
The amount of change in bone loss was measured for a year, and the average value of HD and VD was calculated and graphed. The data of patients who did not return visits were excluded, and the average bone resorption was calculated for one year by dividing the visit time by the non-visit period

The maximum VD was observed in the sixth year, and the largest HD was noted in the second year. Overall, changes of less than 0.1 mm were confirmed (Fig. 6, Tables 1 and 2).

**Table 2:** Average amount of change in bone loss for each year from 1 to 8 years (P < 0.001)

	ΔHD	ΔVD
1Y	− 0.025	− 0.025
2Y	0.090	− 0.057
3Y	− 0.018	0.009
4Y	− 0.032	− 0.063
5Y	0.028	0.030
6Y	0.075	0.093
7Y	0.022	0.075
8Y	0.016	− 0.025

Y, year; HD, Horizontal Bone loss; VD, vertical bone loss;

## Discussion

Despite the limitations inherent in studies with small sample sizes, such as the one investigating marginal bone loss in dental implants, it is often challenging to draw clear conclusions. The absence of a control group in the clinical trial exacerbates these challenges, rendering definitive conclusions elusive.

However, the adoption of a historical control group offers a viable solution to these obstacles. By comparing the outcomes of a previous study, which acts as a historical control, with those of a comparative group that monitors the same patients over an extended period, researchers can effectively assess changes and trends. This approach is particularly advantageous when the data from the prior study is reliable, provides standardized information about the same patient group, and is in alignment with the current research questions and study objectives. Consequently, utilizing a historical control group to compare bone changes after the completion of the first project years constitutes a legitimate methodology (Tables 3 and 4).

**Table 3:** Study population: previous study (control)

No.		Name	Age	Gender	Teeth number	Single/Bridge	Loading	Immediately after				3 Month 1st				3 month 2nd			
								HD1	VD1	HD2	VD2	HD1	VD1	HD2	VD2	HD1	VD1	HD2	VD2
1	S-002	LHK	73	Male	#36	S	I	0.64	0.3	1.31	0.9	0.3	0.77	1	0.87	0.44	0.6	0.6	0.27
2	S-010	JCH	44	Male	#47	S	D	0.2	0.14	0.33	− 0.27	1.65	0.91	1.39	1.21	1.33	0.87	1.18	1.13
3	S-003	LJH	78	Male	#36	S	I	0	− 0.6	0	0	0	− 1.2	0	− 0.41	0	− 0.96	0	− 0.47
4	S-004	JSJ	64	Male	#37	S	D	0.24	0.47	0.11	0.45	0.75	0.54	0.88	0.5	0.65	0.42	1.09	0.78
					#47	S	D	0.42	0.56	0.32	0.21	0.88	0.95	1.05	0.66	1.35	0.77	0.75	0.55
5	S-019	PJM	70	Male	#36	S	D	0.6	1.2	0	1.37	0	0.89	0	1.21	0.4	0.6	0	0
6	S-001	KSD	66	Male	#37	S	D	0	− 0.33	0	− 0.4	0	− 0.28	0	− 0.32	0	− 0.28	0	− 0.32
					#47	S	I	0	− 0.39	0	− 0.24	0	0.3	0	0.27	0	0.3	0	0.27
7	S-014	KSK	76	Female	#47	S	I	0.11	0.12	0.28	− 0.63	0.32	0.49	0.21	0.25	0.67	0.39	0.37	0.2
8	S-006	HYJ	73	Female	#46	B	I	0.29	− 0.53	0.33	− 0.45	0.23	− 0.47	0.21	− 0.34	0.2	0.48	0.21	0.34
					#47		I	0.2	− 0.4	0.27	− 0.7	0.5	0.27	0.14	0.17	0.72	0.75	0.22	0.39
9	S-016	CES	69	Female	#37	S	I	0.27	− 0.44	0.21	0.2	0.48	− 0.42	0.69	0.81	0.34	− 0.35	0.79	0.67
					#47	S	D	0.2	0.19	0.14	0.3	0.1	0.13	0.64	0.37	0.18	0.25	0.24	0.24
10	S-008	PSH	61	Female	#37	S	I	0.3	− 0.34	0.2	0.23	0.2	− 0.33	0.17	0.3	0.41	0.17	0.31	0.3
11	S-018	PTT	39	Female	#36	S	I	0	− 1.21	0	− 0.33	0	− 0.75	0	0	0	− 2.21	0	− 0.34
12	S-011	BHE	57	Female	#35	B	I	0	0	0	0.54	0	0.4	0	0.8	0	0.4	0	1
					#37		I	0	0	0	− 0.27	0	0.1	0	0.7	0	0.67	0	0.6
					#47	S	I	0	0	0	0	0	0	0	0	0	0.1	0	0.1
13	S-012	MKS	66	Female	#45	B	D					1	1.16	0	− 0.5	1.7	1.6	0.68	0.49
					#47		D	0	− 1	0	0.6	0	− 0.81	0	0.76	0	− 1.5	0	0.5
14	S-020	PDA	34	Female	#37	S	I	0.23	− 0.7	0	− 0.74	0.31	− 1.02	0.29	− 0.34	0.14	− 0.51	0.2	− 0.43
15	S-015	YKO	60	Female	#37	S	D	0.29	− 0.42	0.44	0.56	1.15	1.04	1.09	1.05	0.77	0.85	1.12	1.07
					#47	S	D	0.08	0.24	0.31	− 0.27	1.37	1.35	0.98	1.1	1.31	1.49	1.01	1.1

No.		Name	Age	Gender	Teeth number	Single/Bridge	Loading	3 month 3rd				3 month 4th				2Y			
								HD1	VD1	HD2	VD2	HD1	VD1	HD2	VD2	HD1	VD1	HD2	VD2
1	S-002	LHK	73	Male	#36	S	I	0.34	0.77	0.8	0.38	0.61	0.23	0.94	0.43	0.61	0.23	0.94	0.43
2	S-010	JCH	44	Male	#47	S	D	1.47	0.99	1.24	0.97	1.61	0.88	1.35	0.95	1.61	0.88	1.35	0.95
3	S-003	LJH	78	Male	#36	S	I	0	− 0.61	0	− 0.34	0	− 0.81	0	− 0.3	0	− 0.81	0	− 0.3
4	S-004	JSJ	64	Male	#37	S	D	0.79	0.84	0.9	1	0.41	0.24	0.3	0.24	0.41	0.24	0.3	0.24
					#47	S	D	1.38	1.17	1.08	0.88	0.68	0.42	1.12	0.74	0.68	0.42	1.12	0.74
5	S-019	PJM	70	Male	#36	S	D	0.3	0.4	0	0	0	− 0.5	0	0	0	− 0.5	0	0
6	S-001	KSD	66	Male	#37	S	D	0	− 0.28	0	− 0.32	0.2	0.67	0	0.49	0.51	0.7	0.43	0.51
					#47	S	I	0	0.3	0	0.27	0.19	0.45	0.29	0.71	0.29	0.34	0.27	0.7
7	S-014	KSK	76	Female	#47	S	I	0.42	0.2	0.36	0.19	0.42	0.24	0.41	0.23	0.47	0.29	0.44	0.27
8	S-006	HYJ	73	Female	#46	B	I	0.62	0.27	0.15	0.33	0.33	0.33	0.24	0.17	0.33	0.33	0.24	0.17
					#47		I	0.74	1.02	0.27	0.34	0.21	0.24	0.17	0.34	0.21	0.24	0.17	0.34
9	S-016	CES	69	Female	#37	S	I	0.31	0.39	0.56	0.4	0.48	0.17	0.47	0.24	0.48	0.17	0.47	0.24
					#47	S	D	0.41	0.3	0.27	0.18	0.41	0.39	0.49	0.24	0.41	0.39	0.49	0.24
10	S-008	PSH	61	Female	#37	S	I	0.29	0.41	0.41	0.51	0.17	0.41	0.17	0.23	0.17	0.41	0.17	0.23
11	S-018	PTT	39	Female	#36	S	I	0	− 2	0	− 0.34	0	− 2.2	0	− 0.35	0	− 2.2	0	− 0.35
12	S-011	BHE	57	Female	#35	B	I	0	0.54	0	0.88	0	0.6	0	0.68	0	0.4	0	0.54
					#37		I	1.4	1.63	1.76	1.21	1.2	1.47	1.54	1.14	1.74	1.41	1.87	1.27
					#47	S	I	0	0.47	0.4	0.5	0	0.54	0.8	0.5	0	0.81	0	0
13	S-012	MKS	66	Female	#45	B	D	0.72	0	0	− 0.5	0.74	0	0	0	0.74	0	0	0
					#47		D	0	− 1.5	0	0.35	0	− 1.43	0	1.16	0	− 1.43	0	1.16
14	S-020	PDA	34	Female	#37	S	I	0.14	− 0.51	0.2	− 0.43	0.17	− 0.45	0.2	− 0.3	0.17	− 0.45	0.2	− 0.3
15	S-015	YKO	60	Female	#37	S	D	0.77	0.85	1.12	1.07	0.97	1.25	1.1	1.07	0.97	1.25	1.1	1.07
					#47	S	D	1.31	1.49	1.01	1.1	1.32	1.54	1.11	1.34	1.32	1.54	1.11	1.34

		Name	Age	Gender	Teeth number	Single/Bridge	Loading	3Y											
								HD1	VD1	HD2	VD2								
	S-002	LHK	73	Male	#36	S	I	0.61	0.23	0.94	0.43								
	S-010	JCH	44	Male	#47	S	D	1.61	0.88	1.35	0.95								
	S-003	LJH	78	Male	#36	S	I	0	− 0.81	0	− 0.3								
	S-004	JSJ	64	Male	#37	S	D	0.28	0.3	0.48	0.48								
					#47	S	D	0.75	0.64	0.81	0.67								
	S-019	PJM	70	Male	#36	S	D	0	− 0.5	0	0								
	S-001	KSD	66	Male	#37	S	D	0.46	0.71	0.35	0.46								
					#47	S	I	0.31	0.36	0.23	0.59								
	S-014	KSK	76	Female	#47	S	I	0.47	0.29	0.44	0.27								
	S-006	HYJ	73	Female	#46	B	I	0.33	0.33	0.24	0.17								
					#47		I	0.21	0.24	0.17	0.34								
	S-016	CES	69	Female	#37	S	I	0.48	0.17	0.47	0.24								
					#47	S	D	0.41	0.39	0.49	0.24								
	S-008	PSH	61	Female	#37	S	I	0.17	0.41	0.17	0.23								
	S-018	PTT	39	Female	#36	S	I	0	− 2.2	0	− 0.35								
	S-011	BHE	57	Female	#35	B	I	0	0.4	0	0.54								
					#37		I	1.74	1.41	1.87	1.27								
					#47	S	I	0	0.81	0	0								
	S-012	MKS	66	Female	#45	B	D	0.74	0	0	0								
					#47		D	0	− 1.43	0	1.16								
	S-020	PDA	34	Female	#37	S	I	0.15	− 0.22	0.2	− 0.25								
	S-015	YKO	60	Female	#37	S	D	1.02	1.24	1.27	1.17								
					#47	S	D	1.23	1.44	0.99	1.12

**Table 4:** Study population: comparative study

No.		Name	Age	Gender	Teeth number	Single/Bridge	Loading	4Y				5Y				6Y			
								HD1	VD1	HD1	VD1	HD2	VD2	HD2	VD2	HD1	VD1	HD2	VD2
1	S-002	LHK	73	Male	#36	S	I	0.61	0.23	0.61	0.23	0.94	0.43	0.94	0.43	0.61	0.23	0.94	0.43
2	S-010	JCH	44	Male	#47	S	D	1.61	0.88	1.61	0.88	1.35	0.95	1.35	0.95	1.61	0.88	1.35	0.95
3	S-003	LJH	78	Male	#36	S	I	0	− 0.9	0	− 0.9	0	− 0.46	0	− 0.46	0	− 0.9	0	− 0.46
4	S-004	JSJ	64	Male	#37	S	D	0.27	0.37	0.32	0.14	0.68	0.71	0.45	0.25	0.27	0.37	0.45	0.25
					#47	S	D	0.43	0.54	0.62	1.03	0.97	1.05	0.54	0.43	0.43	0.54	0.54	0.43
5	S-019	PJM	70	Male	#36	S	D	0	− 0.5	0	− 0.5	0	0	0	0	0	− 0.5	0	0
6	S-001	KSD	66	Male	#37	S	D	0.46	0.71	0.46	0.71	0.35	0.46	0.35	0.46	0.46	0.71	0.35	0.46
					#47	S	I	0.31	0.36	0.31	0.36	0.23	0.59	0.23	0.59	0.31	0.36	0.23	0.59
7	S-014	KSK	76	Female	#47	S	I	0.47	0.29	0.33	0.14	0.45	0.37	0.44	0.27	0.33	0.14	0.45	0.37
8	S-006	HYJ	73	Female	#46	B	I	0.33	0.33	0.33	0.33	0.24	0.17	0.24	0.17	0.33	0.33	0.24	0.17
					#47		I	0.21	0.24	0.21	0.24	0.17	0.34	0.17	0.34	0.21	0.24	0.17	0.34
9	S-016	CES	69	Female	#37	S	I	0.48	0.17	0.48	0.17	0.47	0.24	0.47	0.24	0.48	0.17	0.47	0.24
					#47	S	D	0.41	0.39	0.41	0.39	0.49	0.24	0.49	0.24	0.41	0.39	0.49	0.24
10	S-008	PSH	61	Female	#37	S	I	0.17	0.41	0.17	0.41	0.17	0.23	0.17	0.23	0.17	0.41	0.17	0.23
11	S-018	PTT	39	Female	#36	S	I	0	− 2.2	0	− 2.2	0	− 0.35	0	− 0.35	0	− 2.2	0	− 0.35
12	S-011	BHE	57	Female	#35	B	I	0	0.4	0	0.4	0	0.54	0	0.54	0	0.4	0	0.54
					#37		I	1.74	1.41	1.74	1.41	1.87	1.27	1.87	1.27	1.74	1.41	1.87	1.27
					#47	S	I	0	0.81	0	0.81	0	0	0	0	0	0.81	0	0
13	S-012	MKS	66	Female	#45	B	D	0.74	0	0.74	0	0	0	0	0	0.74	0	0	0
					#47		D	0	− 1.43	0	− 1.43	0	1.16	0	1.16	0	− 1.43	0	1.16
14	S-020	PDA	34	Female	#37	S	I	0.15	− 0.22	0.15	− 0.22	0.2	− 0.25	0.2	− 0.25	0.15	− 0.22	0.2	− 0.25
15	S-015	YKO	60	Female	#37	S	D	1.05	1.21	1.2	1.34	1.47	1.21	1.34	1.15	1.16	1.29	1.37	1.17
					#47	S	D	1.34	1.39	1.32	1.41	1.18	1.22	1.09	1.17	1.37	1.41	1.12	1.24

No.		Name	Age	Gender	Teeth number	Single/Bridge	Loading	7Y				8Y							
								HD1	VD1	HD2	VD2	HD1	VD1	HD2	VD2				
1	S-002	LHK	73	Male	#36	S	I	0.57	0.3	0.72	0.67	0.47	0.3	0.78	0.44				
2	S-010	JCH	44	Male	#47	S	D	1.61	0.88	1.35	0.95	1.61	0.88	1.35	0.95				
3	S-003	LJH	78	Male	#36	S	I	0	− 0.9	0	− 0.46	0	− 0.9	0	− 0.46				
4	S-004	JSJ	64	Male	#37	S	D	0.32	0.14	0.68	0.71	0.44	0.23	0.77	0.57				
					#47	S	D	0.62	1.03	0.97	1.05	0.72	1.27	1.16	1.06				
5	S-019	PJM	70	Male	#36	S	D	0.28	0	0	0.54	0.28	0	0	0.54				
6	S-001	KSD	66	Male	#37	S	D	0.46	0.71	0.35	0.46	0.46	0.71	0.35	0.46				
					#47	S	I	0.31	0.36	0.23	0.59	0.31	0.36	0.23	0.59				
7	S-014	KSK	76	Female	#47	S	I	0.52	1.23	0.78	1.2	0.54	1.4	0.79	1.15				
8	S-006	HYJ	73	Female	#46	B	I	0.15	0.48	0.34	0.43	0.15	0.48	0.34	0.43				
					#47		I	0.34	0.42	0.23	0.31	0.34	0.42	0.23	0.31				
	S-016	CES	69	Female	#37	S	I	0.48	0.29	0.58	0.23	0.48	0.29	0.58	0.23				
					#47	S	D	0.2	0.5	0.25	0.29	0.2	0.5	0.25	0.29				
10	S-008	PSH	61	Female	#37	S	I	0.31	0.37	0.27	0.51	0.24	0.12	0.55	0.51				
11	S-018	PTT	39	Female	#36	S	I	0	− 2.2	0	− 0.35	0	− 2.2	0	− 0.35				
12	S-011	BHE	57	Female	#35	B	I	0	0.4	0	0.54	0	0.4	0	0.54				
					#37		I	1.74	1.41	1.87	1.27	1.74	1.41	1.87	1.27				
					#47	S	I	0	0.81	0	0	0	0.81	0	0				
13	S-012	MKS	66	Female	#45	B	D	0.74	0	0	0	0.74	0	0	0				
					#47		D	0	− 1.43	0	1.16	0	− 1.43	0	1.16				
14	S-020	PDA	34	Female	#37	S	I	0.19	− 0.26	0.33	0.71	0.19	− 0.26	0.33	0.71				
15	S-015	YKO	60	Female	#37	S	D	1.28	1.33	1.38	1.2	1.25	1.31	1.32	1.18				
					#47	S	D	1.41	1.45	1.27	1.25	1.32	1.41	1.22	1.24				

The methodology of this study, which involves comparing outcomes between a historical control group and a comparison group using the most recent clinical data from KHNMC 2021-01-052-001, collected four years later from the same patients, effectively employs this approach.

The control group study, which aimed to evaluate the clinical outcomes of immediately and early loaded implants with laser-treated surfaces over a three-year period, was based on a previously published article. This control group comprised 15 patients, with a total of 23 implants placed, and successfully concluded the trial without any participants dropping out following the implant surgery.

Hence, despite the challenges posed by a small sample size, leveraging a historical control group provides a justified framework for making claims within a study. This approach not only addresses the limitations associated with small cohorts but also capitalizes on existing, reliable data to substantiate new findings. It highlights the significance of innovative research methodologies in navigating and surmounting the inherent limitations of studies [20].

Historical control groups can be valuable in longitudinal studies that span over a long period of time. By comparing the outcomes of a current group with historical data, researchers can assess changes and trends over time.

Previous study has the following advantage for using as historical control group.

1. Data Quality and Availability: The reliability and availability of historical data are crucial considerations. The patient data were well-recorded and same patient with standardized, and representative of the same population under study. The previous (historical) data aligns with present research question and study objectives.
2. Bias and Confounding Factors: 15 patient population size could be the lack of randomization in previous(historical) control groups, that might introduce the potential for bias and confounding factors. However same patient characteristics, treatment protocols, and there is no other variables between the historical and current groups can affect the validity of the comparison.

The Implant Stability Quotient (ISQ) might have a lower average value at 6 months compared to other observation periods for several reasons. ISQ is utilized to assess the degree of bone integration with the implant, serving as a crucial indicator of the implant's success. A higher ISQ value signifies a more robust connection between the implant and the bone.

In the initial weeks following the placement of the implant, there is a tendency for the ISQ value to rise as the bone around the implant undergoes recovery and strengthening through osseointegration. However, as the osseointegration process stabilizes, there might be a decrease or fluctuation in the ISQ value over time. The 6-month mark may represent a point in time where such changes are noticeable, resulting in a relatively lower average ISQ value compared to other periods.

Studies exploring various surface treatments to enhance osseointegration have contributed to an increased success rate of implants [2, 3, 5, 6]. Sandblasting with large grits and acid-etching (SLA) treated surfaces have demonstrated excellent biocompatibility and affinity for bone [7–10]. The bone-implant contact of SLA surfaces promotes a high degree of osteoblast differentiation, which suggests that these properties of the SLA surface influence its osteoconductive ability [11]. This virtue may reduce loading time and enhance the potential for early loading [11].

Given the significantly superior results of laser-treated surfaces compared to SLA surface implants in a prior animal study [12], we conducted a clinical trial using early loading, which confirmed the previous findings. The application of immediate loading to the implants was determined by assessing the insertion torque at the time of implant placement. In cases of immediate loading, the ISQ values were > 70, indicating that higher initial fixation likely leads to successful outcomes with either immediate or early loading.

The optimal intensity, modality, and duration of laser treatment for dental implant osseointegration vary across the studies. Low-level laser therapy (LLLT) with a wavelength of 940 nm and an output power of 30 mWatts has been used in some studies [1, 2]. Another study used a low-intensity laser with a wavelength of 904 nm and an output power of 20 mW  [3]. The duration of laser treatment ranged from 3 min in three sessions on three alternative days  [4] to 30 s with a dose of 4.7 J/cm^2^  [5]. These laser treatments have shown positive effects on osseointegration, including increased bone density, improved healing capacity, and enhanced secondary stability of dental implants. However, it is important to note that there is still a lack of sufficient case studies, especially in humans, to determine the optimal parameters for laser treatment in dental implant osseointegration. Further research is needed to establish standardized protocols for laser treatment in this context.

Laser treatment of the implant surface rapidly increases the temperature of titanium, causing structural melting, and subsequently increasing the thickness of the oxygen layer [13].

Post-laser treatment, morphological changes and roughness in the titanium become apparent due to the changes in oxygen layer thickness [14]. Laser-treated implants actively promote pre-osteoblast attachment, pre-osteoblastic differentiation, and increased bioactivity [15, 18].

Altered surface roughness aids in adherence of fibrin and migration of osteoblasts, ultimately leading to new bone deposition.

Limitation of this study is as follows;

1. The marginal bone loss of dental implant is relatively complexed issue which is closely related to bone level, screw type, bone defect type, bone filling materials, surgical intervention and et al. It's limitation of this study to make a clear conclusion with the relatively small sample size
2. No supplement information on untreated implants by the same medical team due to insufficient study design is also the limitation of this study.

However, comparing the bone resorption results 3 years after implant placement with the bone changes 5 years later in the same group of patients in the 'old self' study is meaningful. It is difficult to consider the significance of a total of 8 years of long-term bone resorption tracking as insignificant."

Despite differences in observation periods and research methods compared to previous studies, the average annual bone absorption rate of patients after eight years remained at 0.026 mm horizontally and 0.009 mm vertically.

Eight years after implant prosthesis installation, the average value of vertical and horizontal alveolar bone loss was less than 1.5 mm, aligning with the study's objectives.

## Conclusion

Within the constraints of the eight-year retrospective study, the following conclusions can be drawn:

- Laser surface-treated implants with immediate previsualization exhibited radiographic outcomes
- The eight-year follow-up revealed that the marginal bone loss averaged 0.2 mm or less, indicating clinical safety of the laser surface-treated implant system.

## Data Availability

The datasets used and/or analysed during the current study are available from the corresponding author on reasonable request.

## Abbreviations

FBIC: First bone-implant contact
BL: Bone level
SLA: Sandblasting with large grits and acid-etching
HD: Horizontal dimension boneloss
VD: Vertical dimension boneloss
S: Single crown
B: Bridge crown
I: Immediate loading + early loading
D: Delayed loading
TPP: Temporary prosthesis placement
FPP: Final prosthesis placement
